# Factors associated with overall survival, progression-free survival and toxicity in patients with small cell lung cancer and thoracic irradiation in a clinical real-world setting

**DOI:** 10.1186/s13014-023-02252-1

**Published:** 2023-04-18

**Authors:** Marie-Theres Kassik, Dirk Vordermark, Christine Kornhuber, Daniel Medenwald

**Affiliations:** Department of Radiation Therapy, University Hospital Halle/Saale, Ernst-Grube-Str. 40, 06120 Halle, Germany

## Abstract

**Background:**

Small-cell lung cancer (SCLC) is a malignant tumor known for its poor prognosis. In addition to chemotherapy and immunotherapy irradiation plays a big role especially in inoperability. This study evaluated prognostic factors in patients with SCLC, receiving chemotherapy and thoracic irradiation, that may affect overall survival (OS), progression-free survival (PFS) and toxicity.

**Methods:**

Patients with limited disease (LD) SCLC (n = 57) and extensive disease (ED) SCLC (n = 69) who received thoracic radiotherapy were analyzed retrospectively. The prognostic factors sex, age, Karnofsky performance status (KPS), tumor-, nodal-stage and timepoint of start of irradiation in relation to the first cycle of chemotherapy were evaluated. Start of irradiation was stratified as early ($\le$ 2 cycles of chemotherapy), late (3 or 4 cycles) and very late ($\ge$ 5 cycles). Results were analyzed by Cox univariate and multivariate as well as logistic regression analysis.

**Results:**

The median OS of LD-SCLC patients was 23.7 months in early, and 22.0 months in late start of irradiation. In very late start, median OS was not reached. PFS was 11.8, 15.2 and 47.9 months, respectively. In patients with ED-SCLC OS was 4.3 months in early, 13.0 months in late and 12.2 months in very late start of irradiation. PFS was 6.7, 13.0 and 12.2 months, respectively. Prognosis of patients with LD- or ED-SCLC receiving late or very late start of irradiation was significantly prolonged in OS and PFS compared to an early start (*p* < 0.05). KPS $\ge$ 80 shows a significant increase of OS and PFS in ED-SCLC. Female sex and smaller mean lung dose were associated with lower risk of toxicity.

**Conclusion:**

Late or very late start of irradiation is a prognosis-enhancing factor in LD-SCLC and ED-SCLC for OS and PFS. KPS $\ge$ 80 increases prognosis of OS and PFS in ED-SCLC as well. Toxicity is less common in female sex and patients with low mean lung dose in LD-SCLC.

## Introduction

Lung cancer still represents one of the most malignant cancer entities in Germany. In men, it shows the highest cancer mortality and ranks second for incidence, while in women it ranks second for mortality and third for incidence [1].

Among lung cancer subtypes, small cell lung cancer (SCLC) is known for its poor prognosis due to its tendency towards fast proliferation, early dissemination and unspecific or delayed start of symptoms [2]. SCLC is closely associated with heavy smoking [3]. The differentiation between very limited disease (VLD), limited disease (LD) and extensive disease (ED) is relevant for treatment and prognosis. Current curative therapy for patients in LD-SCLC involves a concomitant application of multidrug chemotherapy combining a platinum derivative with etoposide alongside irradiation. Optimal dose and schedule for radiotherapy have not been established. Irradiation can be applied either by hyperfractionation, twice-daily with 1.5 Gy up to a total dose of 45 Gy, or by conventional fractionation once-daily with 1.8 to 2.0 Gy up to a total dose up to 60 or 66 Gy [4]. According to Bonner et al. [5] standard irradiation dose (corresponding to guidelines of 2018) was 2 Gy up to a total dose of 50 Gy to 60 Gy. Studies suggest that in LD-SCLC, irradiation starting within 30 days after the initiation of chemotherapy compared to a later start improves overall survival (OS) and progression-free survival (PFS) [6, 7]. In VLD-SCLC, operative excision of the primary tumor may represent a further therapeutic option. Multidrug chemotherapy with palliative intention is the key therapy in patients with ED-SCLC. In combination with chemotherapy programmed death-ligand 1 (PD-L1)-inhibitors, like atezolizumab or durvalumab, are recommended to prolong OS and PFS [8, 9]. A combination of irradiation and chemotherapy has been associated with improved survival [10], yet different dosages and timing concepts are possible for irradiation. A previous investigation in ED-SCLC compared starting irradiation during the first three cycles of chemotherapy with a later start and detected an insignificantly prolonged OS and PFS in patients who received their first irradiation later than their third cycle of chemotherapy [10]. Strong consent regarding the best time to start irradiation is still lacking in the literature. However, irradiation is usually only practicable after the initiation of chemotherapy in order to reduce tumor volume and irradiation-associated toxicity [11, 12]. In order to lower the incidence of brain metastases, prophylactic cranial irradiation (PCI) is recommended for responding patients in LD-SCLC [13] and those in complete or partial remission after initial chemotherapy in ED-SCLC [3, 14]. In extensive disease with any response to initial chemotherapy and without brain metastasis, periodic contrast-enhanced magnetic resonance imaging (MRI) examination of the brain during follow-up is an alternative to PCI [15]. The estimated median OS was 15–20 months in LD [16] and 8–13 months in ED [17–20] before the introduction of immunotherapy with about 90% of ED-SCLC patients experiencing tumor progression in the first year [21].

Multiple prognostic factors have been analyzed up to date. Age, tumor-, nodes-, metastases- (TNM) stage, tumor markers and inflammatory factors represent relevant indicators connected to OS and PFS [17].

The aim of this study was to identify patient- and therapy-dependent prognostic factors related to OS and PFS under consideration of toxicity.

## Methods

### Data collection

In this retrospective study, all SCLC patients treated with chemotherapy and thoracic radiotherapy at the Department of Radiation Oncology, Martin Luther University Halle-Wittenberg, between January 1, 2015 and December 31, 2019 were enrolled. To differentiate between limited and extensive disease, the classification of Veterans Administration Lung Study Group (VALG) was used. Data collected in this study included sex, age, Karnofsky performance status (KPS), TNM classification, chemotherapy regimens and cycles, thoracic radiation, mean lung dose, planning target volume (PTV), start of thoracic radiation relative to the cycle of chemotherapy, prophylactic cranial irradiation in limited disease, toxicity, response, progression and death. Pulmonaly, gastroenterologic, hematologic and dermatologic toxicities were observed, as well as infections and fatigue. Starting thoracic radiation during the first or second cycle of chemotherapy was defined as “Early start”, and starting radiation during the third or fourth cycle was defined as “Late start”. Radiation starting after the fourth cycle of chemotherapy was defined as “Very late start”. PTV included primary, positron emission tomography-computed tomography (PET-CT) positive lymph-nodes, adjacent lymph-node-stations and a margin of 10–15 mm in all patients. If applicable, additional boost was only applied to the primary tumor and PET-CT-positive lymph nodes.

### Follow up

We collected follow-up information on disease progression until December 31, 2020 and on overall survival until December 31, 2021. Progression was diagnosed by a radiograph or computer tomography (CT) scan as well as a cranial MRI. Registration offices collected dates of death. We defined overall survival (OS) as the time between the date of diagnosis and the date of death or last day of follow-up. Progression free survival (PFS) describes time from the date of diagnosis until the date of progression, date of death or last day of follow-up. Patients lost to follow up were declared as censored. We excluded patients with missing information on death from OS analyses.

### Data analysis

The impact of positive prognosis factors for OS and PFS was estimated by Cox proportional hazards model applying univariate analyses for sex, age, KPS, T-stage, N-stage and start of thoracic radiation relative to the cycle of chemotherapy. Multivariate Cox regression was performed for parameters that showed a statistically significant impact (*p* < 0.05) in univariate analyses to detect independent prognostic factors.

Existence of at least one toxicity (pneumological, gastroenterological, neurological, hematological, dermatological or psychological) in therapy regimes of patients in LD-SCLC, as well as the occurrence of pneumonitis were analyzed by logistic regression analysis, adjusted for age, sex, KPS, start of radiation, PTV and mean lung dose. Statistical analyses were performed by SPSS statistics 27.0 software.

## Results

### Patient characteristics

A total of 126 patients were treated at the Department of Radiation Oncology, Martin Luther University Halle-Wittenberg, between January 1, 2015 and December 31, 2019 and were enrolled in the study. Fifty-seven of these patients were in LD-SCLC, while 69 patients were in ED-SCLC. All patients received conventionally fractionated irradiation once daily. Date of death was missing for two patients with LD and three patients with ED. These patients were excluded from subsequent analyses of OS. Further categorization considered the start of irradiation. In the LD group, information about toxicity was missing for one patient who was subsequently excluded from analysis of toxicity. Treatment concepts contained various schedules of TRT, chemotherapy and antibodies (atezolizumab, nivolumab, bevacizumab, pembrolizumab, rovalpituzumab).The characteristics of the 57 patients in LD (Table 1) and 69 patients in ED (Table 2) are presented according to timepoint of irradiation.

**Table 1:** Patient characteristics in LD-SCLC according to timepoint of start of irradiation

Limited disease (57)	Early start 24	Late start 25	Very late start 8
*Sex*			
Male	16 (66.67)	8 (32.00)	5 (62.50)
Female	8 (33.32)	17 (68.00)	3 (37.50)
*Age*			
Average	65.36	64.79	67.81
Range	45–78	49–70	60–84
*Karnofsky performance status*			
Median	80	70	80
50–70	11 (45.83)	14 (56.00)	2 (25.00)
80–100	13 (54.17)	11 (44.00)	6 (75.00)
*Chemotherapy regimens*			
CE (platinum derivatives/etoposide)	13 (54.17)	18 (72.00)	5 (62.50)
CEA (platinum derivatives/etoposide/ antibodies)	3 (12.50)	4 (16.00)	0 (0.00)
Others	8 (33.33)	3 (12.00)	3 (37.5)
*Time to irradiation*			
Median in days	43	75	139.5
Range	21–105	45–152	83–329
*Concepts of doses*			
2 Gy up to 50 Gy	1 (4.17)	11 (44.00	2 (25.00)
2 Gy up to 50 Gy + 10 Gy Boost	16 (66.67)	14 (56.00)	4 (50.00)
Others	7 (29.17)	0 (0.00)	2 (25.00)
*Planning Target Volume in cm*^*3*^			
Median	684.67	614.00	670.54
Range	7.78–2239.29	245.91–1905.74	134.18–1002.61
*Mean lung dose in Gy*			
Median	13.78	15.22	14.69
Range	1.42–19.49	10.25–22.39	7.64–18.68
*Mean lung volume in ccm*			
Median	3645.94	3625.67	3046.75
Range	1960.39–5191.14	1856.19–6143.98	2354.10–4517.23
*V5 in ccm*			
Median	2170.78	2144.41	1729.40
Range	981.7–3779.65	1093.65–4235.21	1453.84–2519.97
*V20 in ccm*			
Median	925.15	1029.65	934.38
Range	634.48–1695.67	437.77–1625.65	653.2–1162.65
*Mean heart dose in Gy*			
Median	19.81	20.00	13.24
Range	6.91–29.99	3.49–28.58	4.12–34.52
*Maximum dose esophagus in Gy*			
Median	50.9	51.1	50.6
Range	20.4–52.6	42.00–55.00	26.9–52.6
Prophylactic cranical irradiation	5 (20.83)	3 (12.00)	3 (37.50)
2 Gy up to 30 Gy	5 (20.83)	2 (8.00)	3 (37.50)
others	0 (0.00)	1 (4.00)	0 (0.00)
Toxicity at least 1	16 (66.67)	17 (68.00)	6 (75.00)
Pneumonitis	3 (12.50)	7 (28.00)	3 (37.50)
*Remission status after thoracic radiation*			
Not stated	1 (4.17)	1 (4.00)	0 (0.00)
Stable	2 (8.34)	2 (8.00)	2 (25.00)
Partial remission	12 (50.00)	12 (48.00)	3 (37.50)
Complete remission	4 (16.67)	7 (28.00)	2 (25.00)
Progressive disease	5 (20.83)	3 (12.00)	1 (12.50)
*Progression-free survival (PFS) in months*			
Median after date of diagnosis	11.8 (4.7–18.8)	15.2 (7.3–23.2)	47.9 (0.0–97.2)
Median after thoracic radiation	7.5 (1.6–13.4)	11.9 (3.1–20.6)	9.4
*Overall survival (OAS) in months*			
Median after date of diagnosis	23.7 (16.6–30.8)	22.0 (15.8–28.2)	–
Median after thoracic radiation	22.3 (13.5–31.1)	18.5 (11.5–25.6)	–
2-year overall survival rate after date of diagnosis	10 (41.67)	13 (52.00)	4 (50.00)

**Table 2:** Patient characteristics in ED-SCLC according to timepoint of start of irradiation

Extensive disease (69)	Early start 7	Late start 6	Very late start 56
*Sex*			
Male	5 (71.43)	5 (83.33)	35 (62.50)
Female	2 (28.57)	1 (16.67)	21 (37.50)
*Age*			
Average	63.99	49.96	63.91
Range	52–76	38–68	39–85
*Karnofsky performance status*			
Median	70	85	70
50–70	5 (71.43)	2 (33.33)	36 (64.29)
80–100	2 (28.57)	4 (66.67)	20 (35.71)
*Chemotherapy regimens*			
CE (platinum derivatives/etoposide)	6 (85.71)	3 (50.00)	33 (58.93)
CEA (platinum derivatives/etoposide/ antibodies)	1 (14.29)	2 (33.33)	8 (14.29)
Others	0 (0.00)	1 (16.67)	15 (26.79)
*Time to irradiation*			
Median in days	30	83	183
Range	14–181	43–398	79–553
*Concepts of doses*			
2 Gy up to 50 Gy	1 (14.29)	2 (33.33)	24 (42.86)
2 Gy up to 50 Gy + 10 Gy Boost	2 (28.57)	2 (33.33)	7 (12.50)
2.5 Gy up to 40 Gy	0 (0.00)	0 (0.00)	9 (16.07)
2.5 Gy up to 50 Gy	1 (14.29)	1 (16.67)	10 (17.86)
Others	4 (56.58)	1 (16.67)	19 (33.93)
*Mean lung dose in Gy*			
Median	10.92	16.12	13.47
Range	1.72–14.58	9.95–23.04	2.90–21.44
Prophylactic cranical irradiation	0 (0.00)	0 (0.00)	0 (0.00)
Toxicity at leats 1	4 (56.58)	6 (100.00)	40 (71.43)
*Remission status after thoracic radiation*			
Not stated	0 (0.00)	0 (0.00)	6 (10.71)
Stable	0 (0.00)	0 (0.00)	3 (85.36)
Partial remission	5 (41.43)	5 (83.33)	14 (25.00)
Complete remission	0 (0.00)	0 (0.00)	6 (10.71)
Progressive disease	2 (28.57)	1 (16.67)	27 (48.21)
*Progression-free survival (PFS) in month*			
Median after date of diagnosis	6.4 (3.6–9.1)	13.0 (6.9–19.1)	12.2 (9.6–14.7)
Median after thoracic radiation	3.1 (0.0–7.6)	2.7 (0.0–10.0)	2.3 (1.4–3.3)
*Overall survival (OAS) in month*			
Median after date of diagnosis	4.3 (0.0–11.7)	39.4 (0.0–92.8)	12.1 (11.2–13.0)
Median after thoracic radiation	1.4 (0.0–4.0)	31.5 (0.0–93.3)	4.6 (3.8–5.5)
2-year overall survival rate after date of diagnosis	0 (0.00)	3 (50.00)	5 (8.93)

### Prognostic analyses of overall survival and progression-free survival in LD-SCLC

**Fig. 1:**
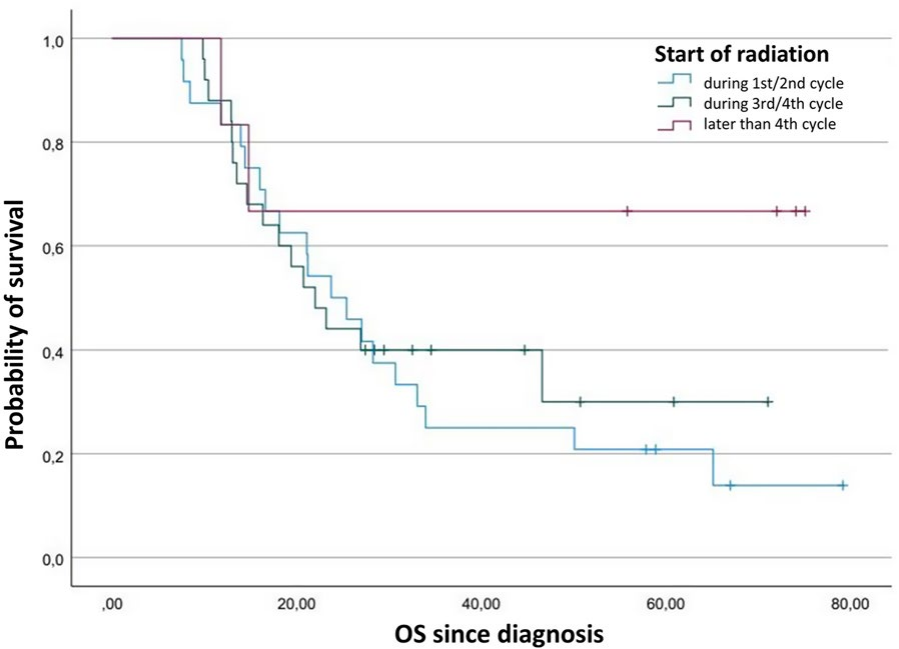
Kaplan–Meier plot comparing the overall survival function after diagnosis depending on timepoint of start of radiation in LD-SCLC

**Fig. 2:**
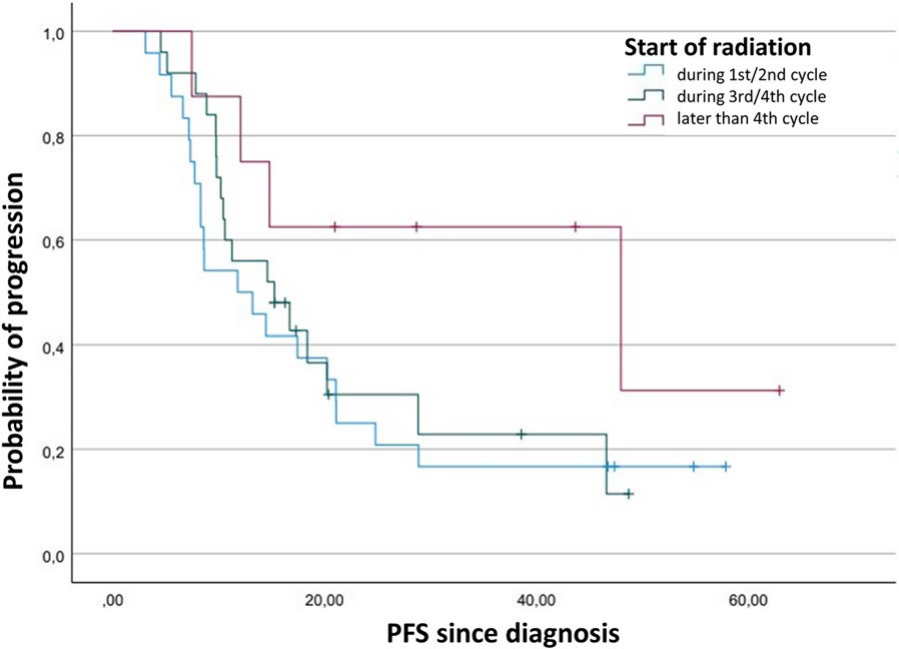
Kaplan–Meier plot comparing the progression-free survival function after diagnosis depending on timepoint of start of radiation in LD-SCLC

**Fig. 3:**
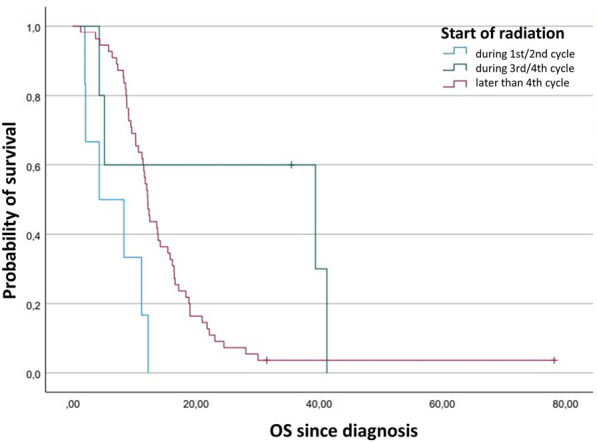
Kaplan–Meier plot comparing the overall survival function after diagnosis depending on timepoint of start of radiation in ED-SCLC

**Fig. 4:**
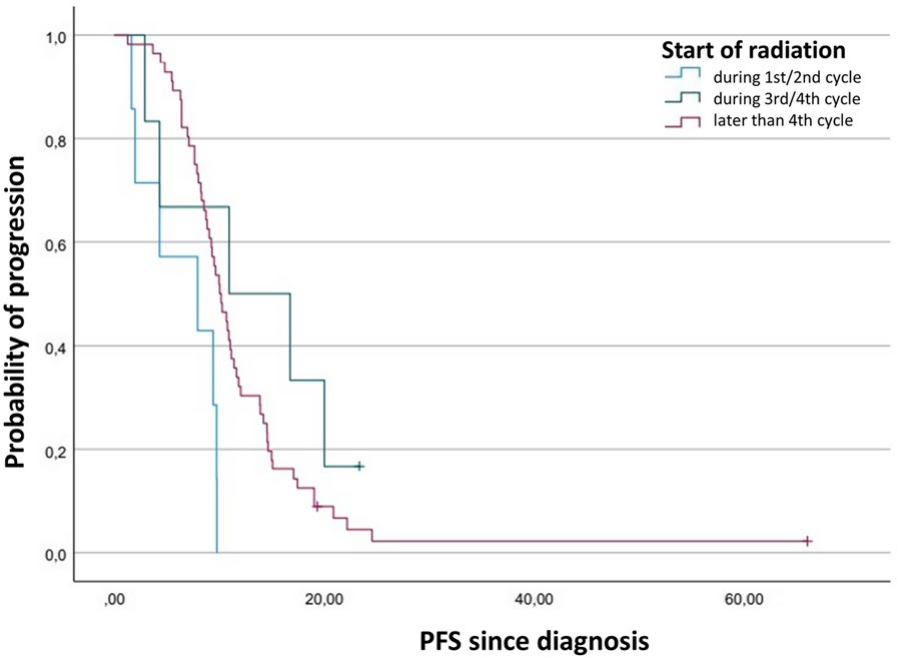
Kaplan–Meier plot comparing the progression-free survival function after diagnosis depending on timepoint of start of radiation in ED-SCLC

Fifty-five patients were included in this analysis of OS. Median OS after date of diagnosis in patients receiving thoracic irradiation during the first or second cycle of chemotherapy was 23.7 months (95% confidence interval (CI) 16.6–30.8). Receiving thoracic radiotherapy (TRT) during the third or fourth cycle resulted in a median OS of 22.0 months (95% CI 15.8–28.2). The median for the start of irradiation later than the fourth cycle was not reached till the end of follow-up (Fig. 1). Multivariate analyses displayed a statistically insignificant hazard ratio of 0.9 (95% CI 0.4–2.0) for a late start (*p* = 0.66) and 0.2 (95% CI 0.0–1.1) (*p* = 0.06) for a very late start, each compared to an early start of irradiation. In the analysis of PFS, 57 patients were included. Median PFS after the date of diagnosis in patients who were irradiated at early start was 11.8 months (95% CI 4.7–18.8), at late start it was 15.2 months (95% CI 7.3–23.2) and at very late start it was 47.9 months (95% CI 0.0–97.2) (Fig. 2). Multivariate analysis detected a significant impact of start of radiation with a hazard ratio of 0.3 (95% CI 0.0–1.0) for very late start in relation to early start (Table 3) (Figs. 3, 4).

**Table 3:** Analysis of prognostic factors influencing overall survival and progression-free survival since diagnosis in LD-SCLC

Characteristics	OS since diagnosis				PFS since diagnosis			
	Univariate		Multivariate		Univariate		Multivariate	
	HR (95% CI)	*p*	HR (95% CI)	*p*	HR (95% CI)	*p*	HR (95% CI)	*p*
Sex								
Male	Ref.				Ref.			
Female	0.7 (0.4–1.3)	0.23	0.8 (0.4–1.8)	0.63	0.7 (0.4–1.3)	0.22	0.6 (0.3–1.3)	0.22
Age	1.0 (1.0–1.1)	0.74	1.0 (1.0–1.1)	0.81	1.0 (1.0–1.0)	0.90	1.0 (1.0–1.0)	0.87
Karnofsky								
80–100	Ref.				Ref.			
< 80	1.5 (0.8–2.9)	0.19	1.4 (0.7–3.0)	0.37	1.1 (0.6–2.1)	0.69	0.7 (0.3–1.6)	0.46
T-Classification								
T1	Ref.				Ref.			
T2	0.8 (0.2–2.9)	0.75	0.6 (0.1–2.7)	0.48	1.4 (0.4–5.0)	0.58	1.3 (0.3–5.9)	0.75
T3	0.5 (0.2–1.6)	0.28	0.5 (0.2–1.8)	0.32	0.8 (0.3–2.4)	0.65	0.8 (0.2–3.0)	0.79
T4	0.9 (0.3–2.1)	0.73	0.6 (0.2–1.7)	0.31	1.8 (0.7–4.9)	0.24	1.6 (0.5–5.2)	0.41
N-Classification								
N0	Ref.				Ref.			
N1	0.5 (0.1–2.5)	0.38	0.9 (0.2–5.6)	0.95	0.5 (0.1–1.8)	0.26	0.5 (0.1–2.4)	0.40
N2	1.4 (0.5–3.7)	0.53	1.9 (0.6–5.9)	0.24	0.9 (0.4–2.3)	0.86	0.8 (0.3–2.2)	0.63
N3	1.4 (0.5–3.8)	0.56	2.6 (0.7–9.2)	0.14	1.4 (0.5–3.5)	0.54	1.5 (0.5–4.6)	0.50
Start during cycle								
Early start	Ref.				Ref.			
Late start	0.9 (0.4–1.7)	0.66	0.9 (0.4–2.0)	0.73	0.8 (0.4–1.6)	0.62	1.0 (0.5–2.2)	0.99
Very late start	0.3 (0.1–1.3)	0.10	0.2 (0.0–1.1)	0.06	0.4 (0.1–1.2)	0.09	0.3 (0.1–1.0)	0.05

### Sensitivity analysis LD-SCLC

**Fig. 5:**
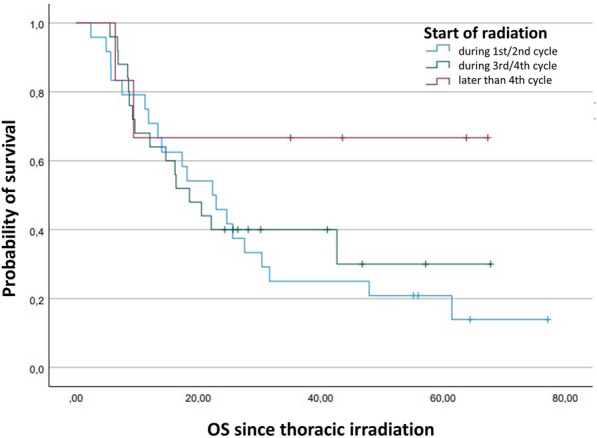
Kaplan–Meier plot comparing the overall survival function after thoracic irradiation depending on timepoint of start of radiation in LD-SCLC

**Fig. 6:**
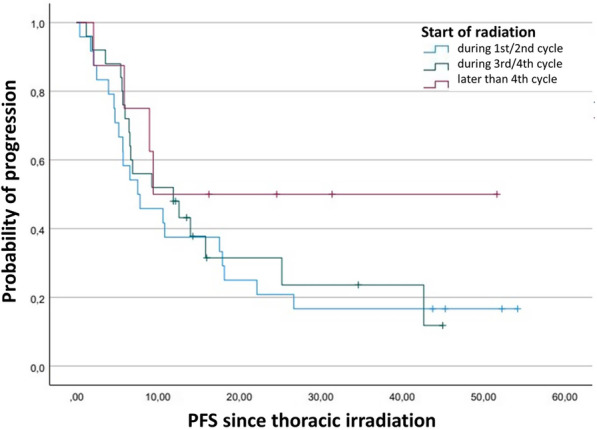
Kaplan–Meier plot comparing the progression-free survival function after thoracic irradiation depending on timepoint of start of radiation in LD-SCLC

By changing startpoint of analysis for OS and PFS from date of diagnosis to the last day of first thoracic irradiation, output variable HR shows almost identical values in univariate and multivariate analyses Evaluations of prognostic factors of OS and PFS after first irradiation are shown in “Table 7 of the Appendix”. Kaplan–Meier curves of OS and PFS after thoracic irradiation are shown in Figs. 5 and 6.

### Prognostic analyses of overall survival and progression-free survival in ED-SCLC

In the univariate and multivariate analyses of 66 patients, a statistically significant impact on OS could be shown for KPS with a hazard ratio of 2.4 (95% CI 1.3–4.5). In relation to early start irradiation, a significant hazard ratio in patients with late start of irradiation was 0.1 (95% CI 0.0–0.5), and in patients with very late start 0.2 (95% CI 0.0–0.7). Median OS after date of diagnosis was 4.3 (95% CI 0.0–11.7) months in patients with early start of irradiation, 39.4 (95% CI 0.0–92.8) months in patients with late start and 12.1 (95% CI 11.2–13.0) months in patients with very late start of irradiation. In the univariate Cox regression analysis of 69 patients, a significant impact on PFS with a hazard ratio of 1.9 (95% CI 1.2–3.2) resulted for KPS 80–100 in comparison to KPS $<$ 80. With regards to the start of thoracic radiation in relation to chemotherapy, the results for the endpoint PFS remained statistically significant in univariate analyses. The hazard ratio in patients with late start of irradiation was 0.2 (95%CI 0.1–0.6) and in patients with very late start of irradiation it was 0.3 (95% CI 0.1–0.7). Multivariate analysis yielded hazard ratios of 0.3 (95% CI 0.1–1.4) in patients with late start of irradiation (*p* = 0.14) and 0.4 (95% CI 0.1–1.1) in patients with very late start (*p* = 0.09), each in relation to early start (Fig. 4). Median PFS after the date of diagnosis was 6.4 (95% CI 3.6–9.1) months in patients with early start of irradiation, 13.0 (95% CI 6.9–19.1) months in patients with late start and 12.2 (95% CI 9.6–14.7) months in patients with very late start (Table 4).

**Table 4:** Analysis of prognostic factors influencing overall survival and progression-free survival since diagnosis in ED-SCLC

Characteristics	OS since diagnosis				PFS since diagnosis			
	Univariate		Multivariate		Univariate		Multivariate	
	HR (95% CI)	*p*	HR (95% CI)	*p*	HR (95% CI)	*p*	HR (95% CI)	*p*
Sex								
Male	Ref.				Ref.			
Female	1.0 (0.6–1.6)	0.92	1.0 (0.5–1.8)	0.99	1.0 (0.6–1.6)	0.94	1.1 (0.6–2.0)	0.73
Age	1.0 (1.0–1.0)	0.27	1.0 (1.0–1.0)	0.61	1.0 (1.0–1.0)	0.67	1.0 (1.0–1.0)	0.55
Karnofsky								
80–100	Ref.				Ref.			
< 80	2.7 (1.6–4.7)	< 0.001	2.4 (1.3–4.5)	0.01	1.9 (1.2–3.2)	0.01	1.7 (0.9–3.0)	0.10
T-Classification								
T1	Ref.				Ref.			
T2	0.6 (0.2–1.9)	0.36	0.9 (0.2–4.8)	0.93	0.3 (0.1–1.1)	0.06	0.3 (0.1–1.4)	0.13
T3	1.1 (0.3–3.5)	0.88	1.6 (0.4–6.9)	0.53	0.6 (0.2–2.0)	0.43	0.7 (0.2–2.4)	0.53
T4	0.8 (0.3–2.3)	0.66	1.2 (0.3–5.5)	0.78	0.6 (0.2–1.7)	0.31	0.5 (0.1–1.8)	0.28
N-Classification								
N0	Ref.				Ref.			
N1	0.2 (0.0–2.4)	0.23	1.3 (0.1–21.7)	0.85	0.2 (0.0–1.8)	0.15	0.4 (0.0–5.0)	0.44
N2	1.2 (0.2–9.3)	0.84	3.7(0.3–41.3)	0.29	0.7 (0.1–5.3)	0.73	1.2 (0.1–12.4)	0.87
N3	0.7 (0.1–5.4)	0.76	3.4 (0.3–41.0)	0.34	0.7 (0.1–5.2)	0.74	1.5 (0.1–15.5)	0.74
Start during cycle								
Early start	Ref.				Ref.			
Late start	0.1 (0.0–0.4)	0.001	0.1 (0.0–0.5)	0.01	0.2 (0.1–0.6)	0.01	0.3 (0.1–1.4)	0.14
Very late start	0.3 (0.1–0.6)	0.003	0.2 (0.0–0.7)	0.01	0.3 (0.1–0.7)	0.01	0.4 (0.1–1.1)	0.09

### Sensitivity analysis ED-SCLC

**Fig. 7:**
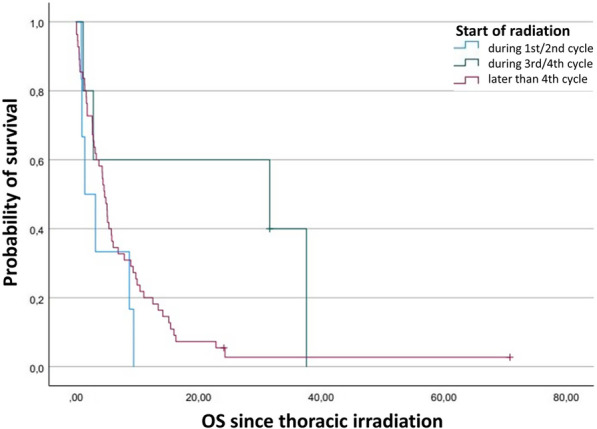
Kaplan–Meier plot comparing the overall survival function after thoracic irradiation depending on timepoint of start of radiation in ED-SCLC

**Fig. 8:**
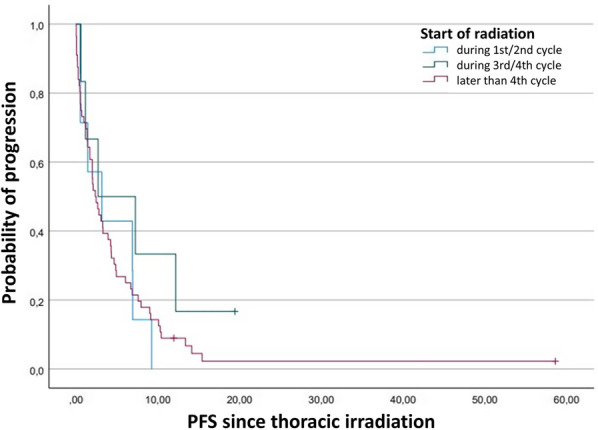
Kaplan–Meier plot comparing the progression-free survival function after thoracic irradiation depending on timepoint of start of radiation in ED-SCLC

By changing the input variable from OS since diagnosis to OS from the last day of first thoracic irradiation in univariate and multivariate analyses, the output variable HR showed the same trend. In the sensitivity analysis for PFS, by changing from PFS since diagnosis to PFS from the last day of first thoracic irradiation, the trend of HR remained similar in univariate and multivariate analysis of sex and KPS but differed in T-stadium, N-stadium and timepoint of start of radiation. Evaluations of prognostic factors of OS and PFS after first irradiation are shown in “Table 8 of the Appendix”. Kaplan–Meier curves of OS and PFS after first irradiation are shown in Figs. 7 and 8.

### Toxicity in LD-SCLC

Toxicity in the upper gastrointestinal tract was reported most frequently (28), pneumological (13), hematological (12) and dermatological (10) side effects were also common. To detect factors influencing possible side effects after irradiation, a logistic regression analysis was carried out for the variables age, sex, KPS, start of TRT according to chemotherapy, mean lung dose and PTV. Fifty-six patients were enrolled in this analysis. Statistically significant impact was detected in female patients in relation to male patients and mean lung dose with odds ratios (OR) of 0.2 (95% CI 0.0–1.0) and 1.4 (95% CI 1.1–1.8), respectively (Table 5).

**Table 5:** Analysis of factors influencing the occurrence of toxicities patients with radiochemotherapy in LD-SCLC

Characteristics	Toxicity (at least 1)	
	OR (95% CI)	*p*
Age	1.0 (0.9–1.1)	0.45
Sex		
Male	Ref.	
Female	0.2 (0.0–1.0)	0.04
Karnofsky		
80–100	Ref.	
< 80	1.0 (0.3–4.1)	0.96
Start during cycle		
Early start	Ref.	
Late start	0.9 (0.2–4.5)	0.94
Very late start	1.0 (0.1–9.0)	0.98
Mean lung dose	1.4 (1.1–1.8)	0.01
PTV	1.0 (1.0–1.0)	0.17

Most common side effect was pneumonitis. In order to identify whether the characteristics have an influence on the occurrence of the side effect pneumonitis, the analysis for this outcome variable was carried out again. In female patients in relation to male patients odds ratio of 0.2 (95% CI 0.0–1.2) was detected. Odds ratio in late start of irradiation and very late start of irradiation were 3.8 (95% CI 0.7–22.1) and 4.9 (95% CI 0.6–40.0). This results were not significant as well. Results are shown in Table 6.

**Table 6:** Analysis of factors influencing the occurrence of pneumonitis patients with radiochemotherapy in LD-SCLC

Characteristics	Pneumonitis	
	OR (95% CI)	*p*
Age	1.0 (0.9–1.1)	0.45
Sex		
Male	Ref.	
Female	0.2 (0.0–1.2)	0.08
Karnofsky		
80–100	Ref.	
< 80	1.3 (0.3–5.6)	0.72
Start during cycle		
Early start	Ref.	
Late start	3.8 (0.7–22.1)	0.14
Very late start	4.9 (0.6–40.0)	0.14
Mean lung dose	1.2 (0.9–1.6)	0.15
PTV	1.0 (1.0–1.0)	0.44

## Discussion

This study analyzed 57 patients in LD-SCLC and 69 patients in ED-SCLC. Results suggest that in radiochemotherapy of SCLC the timing of irradiation was associated with differences in overall survival and progression-free survival. Despite the partial lack of significance, it was identified that in LD-SCLC very late start of irradiation is superior to early start with respect to OS and PFS. In contrast, no superiority of early or late start of irradiation could be established in terms of OS and PFS. Toxicity in LD-SCLC was dependent on sex and mean lung dose. Analysis of the ED-SCLC group showed that starting irradiation after the second cycle of chemotherapy is associated with prolonged OS and PFS. This was the case for patients with Karnofsky performance status $\ge$ 80 as well.

For LD-SCLC, existing data concerning the impact of start of radiation on OS remain inconclusive. The results of Wang et al. [22] confirmed superiority in OS and response of patients with complete or partial remission after two or three cycles of initial chemotherapy. Further studies have shown superiority of an early start of thoracic radiation over a late or very late start. Patients receiving irradiation during the first or second cycle of chemotherapy presented prolonged median OS [7, 23], PFS [23], 2-, 3 and 5-year OS [7, 24] and better local control [24].

However, when one of these studies was replicated, an increased OS in patients receiving radiation concurrently with their sixth cycle of chemotherapy compared to those receiving radiation concurrently with their first cycle (15.1 months vs 13.7 months) was reported [25]. However, due to the wide CI range of 0.72 to 1.28 results were deemed as insignificant. In contrast, the working group of Perry et al. found significantly increased rates of complete remission and 2-year OS as well as 2-year-failure-free-OS in the group with late start of radiation (starting during the fourth cycle of chemotherapy vs starting during the first cycle) [26]. However, treatments used in this study are hardly comparable to therapies applied in the clinical setting today. Other publications on this subject were unable to detect differences in OS and incidence of recurrence and presented no recommendation on the timing of irradiation [27, 28]. Our study identified increased OS and PFS in patients beginning irradiation after the start of the fourth cycle of chemotherapy. Although only multivariate analysis of PFS was significant, all results indicated the trend of superiority of very late start compared with early and late start of radiation in terms of OS and PFS. This could be explained by the fact that SCLC is very chemotherapy-sensitive [29]. After initial volume reduction by chemotherapy, radiation can target the reduced tumor tissue more intensively. It is safe to administer several courses of chemotherapy before applying radiotherapy. In addition, it should be considered whether the patients receiving late TRT were in better health conditions at the time of diagnosis. This could have led to the radiation being delayed even further or only during a recurrence. Therefore, these patients had a better chance of long-term OS and PFS from the start. Although, the sensitivity analysis performed in this study showed similar values in OS and PFS after diagnosis compared to OS and PFS after first irradiation, which suggests this bias can be objected. But it must be noted that due to the wide confidence interval in these results, a negative effect on OS and PFS cannot be ruled out. Previous publications have already identified further prognostic factors associated with prolonged OS, like female sex [30–33], KPS ≥ 70 [34] respectively Eastern Cooperative Oncology Group (ECOG) performance status 0–1 [33, 35] or N-Stage 0–2 [34] as well as therapy-related factors like radiation dose > 52 Gy [34] or PCI [36]. In our analysis, a trend of protective influence of female sex, KPS 80–100 in OS were detected but insignificant.

Since the influence of mean lung dose on toxicity dose was only significant after adjusting for PTV, it can be concluded that the TRT dose to the lung irrespective of the PTV is disadvantageous, while the contribution of the PTV itself to the lung dose is of little effect in terms of toxicity. Timing of irradiation had no influence on toxicity, a consistent finding with results of other publications [24, 27]. Takada et al. [7] suggested an increased hematological toxicity and esophagitis in patients with an early start of irradiation compared to a late start treatment. It should be mentioned that the frequency of chemotherapy varied in the study. Patients with early start TRT received cytostatics in an interval of three weeks and patients with late start TRT in an interval of four weeks. This may have an impact especially on hematological toxicity. Another study supported the results of Takada et al. by detecting increased rates of neutropenia in patients undergoing a simultaneous start of chemotherapy and radiation compared to patients starting radiation after three cycles of chemotherapy [26]. As demonstrated by Singh et al. [31] toxicity could also depend on sex. In their analysis women were more likely to suffer from hematological and gastrointestinal side effects like vomiting and stomatitis as well as infection. Our study also identified sex as a factor influencing toxicity, but in contrast to Singh et al. different toxicities were observed. Unlike in the study by Singh et al., female sex is identified as protective factor in this analysis. Analysis of outcome variable occurrence of pneumonitis detected an insignificant trend of female sex and lower mean lung dose as protective factors as well. However, although insignificant, late and very late start of irradiation appeared to be associated with an increased incidence of pneumonitis. This could be explained by the fact of a higher cumulative dose of chemotherapy at the start of radiation.

Skarlos et al. [28] compared start of irradiation with the first or with the fourth cycle of chemotherapy with the similar result of an increased occurrence of pneumonitis in patients with the late start of irradiation (not significant).

For ED-SCLC a role of thoracic irradiation is still controversial. As demonstrated by Slotman et al. [37] thoracic radiotherapy in addition to PCI is recommended in every patient with response after initial chemotherapy. Although they could not prove increased 1-year OS, 2-year OS was significantly prolonged. After TRT tumor progression was less likely and six months after radiotherapy PFS was better in the irradiated group than in the control group. As reported by further publications, it is recommended that TRT should be added to chemotherapy to reduce local recurrence [38] and to prolong survival [24, 39]. Shang et al. [40] detected that in ED-SCLC patients with distant metastasis, TRT improves OS, especially in those with only one metastatic site.

However, evidence of timing of TRT in ED-SCLC is still insufficient. The present study shows increased OS in patients receiving TRT during the third cycle of initial chemotherapy or later. This can also be explained by chemotherapy-sensitive SCLC [29]. Effective chemotherapy initially often results in rapid responses and noticeable improvement in symptoms [41]. The tumor tissue should primarily react to cytostatics. Since ED-SCLC is not entirely located in the lungs, but also in nodal and distant organ metastases, chemotherapy plays a bigger role than TRT at the beginning of therapy. This allows the TRT to have a more intensive effect on the remaining tissue. While mean OS was 8 to 13 months [17–20] in the pre-immunotherapy era, 39.4 months in late start irradiation represents a significant increase, but the wide 95% CI for OS in late start from 0.0 to 92.8 must be mentioned. The sensitivity analysis shows that good OS in patients with late start is not affected by the fact that the patient may be in a better health condition at the date of diagnosis receiving late TRT and patients in worse health conditions start radiation earlier, because OS after first irradiation give similar values. Values in PFS differ by T-stage, N-stage and timepoint of start of radiation. A possible explanation is that patients with a small tumor or low metastatic tendency are irradiated later on, or only after the tumor begins progression, and so they tend to continue to experience tumor progression immediately after their first irradiation, however, overall they have better chances for long PFS after diagnosis.

Although the influence on OS could be proven, evidence for prolonged PFS was only significant in univariate analysis but with a trend identified in multivariate analysis. In contrast to LD-SCLC, there was only one more study investigating the impact of timing of TRT in ED-SCLC. Luo et al. [10] defined early TRT as irradiation during the third cycle of chemotherapy or earlier and compared it to late TRT. Despite lacking significance, improved OS and PFS of patients receiving late TRT and in contrast, better locoregional recurrence-free survival of patients receiving early TRT was detected.

Another beneficial prognostic factor in ED-SCLC is Karnofsky performance status ≥ 80, influencing both OS and PFS, as demonstrated in this study. Further publications confirmed this factor or the equivalent ECOG 0–1. [32, 33, 35, 42] Although an advantage in OS of female sex could not be determined in this study, it has already been reported in other publications [31, 33]. Previously recognized harmful prognostic factors are tumor-related, including large tumor size, multiple metastatic sites at diagnosis [43] and patient-related factors such as smoking index ≥ 400 (number of cigarettes smoked per day * years of tobacco smoking) and age ≥ 70 [17, 44].

The retrospective nature of this analysis is its major limitation, leading to a lack of unity in chemotherapy regimens, dose and target volumes. In ED, multidrug chemotherapy includes different types of cytostatics and current immunotherapy as well as second line chemotherapy. Patients received chemotherapy over the course of five years, however, during this time the therapy recommendations have changed. All data were collected from clinical documents by referring hospitals. Some information on psychological toxicity, such as fatigue, is dependent on subjective assessment of patients. In addition, 126 patients were divided into two analysis groups (LD and ED), which therefore represented limited numbers and would need to be expanded to confirm the results. Differences in radiation dose, methods and chemotherapy and immunotherapy regimens can contribute to the bias of this study.

## Conclusion

In conclusion, in ED-SCLC starting TRT following the start of the first or second cycle of first-line chemotherapy is associated with increased OS. A further prognostic factor relating to extended OS and PFS is KPS $\ge$ 80. In LD-SCLC, starting radiation later than the fourth cycle of chemotherapy specifically prolonged PFS. Furthermore, toxicity in LD-SCLC was found to be influenced by sex and mean lung dose.

## Data Availability

The datasets generated during the current study are available from the corresponding author on reasonable request.

## Appendix

See Figs. 5, 6, 7 and 8, Tables 7 and 8

**Table 7:** Analysis of prognostic factors influencing overall survival and progression-free survival since thoracic irradiation in LD-SCLC

Characteristics	OS since thoracic radiation				PFS since thoracic radiation			
	Univariate		Multivariate		Univariate		Multivariate	
	HR (95% CI)	*p*	HR (95% CI)	*p*	HR (95% CI)	*p*	HR (95% CI)	*p*
Sex								
Male	Ref.				Ref.			
Female	0.7 (0.4–1.3)	0.23	0.8 (0.4–1.7)	0.53	0.7 (0.4–1.3)	0.25	0.6 (0.3–1.2)	0.16
Age	1.0 (1.0–1.1)	0.59	1.0 (1.0–1.1)	0.56	1.0 (1.0–1.1)	0.67	1.0 (1.0–1.0)	0.55
Karnofsky								
80–100	Ref.				Ref.			
< 80	1.6 (0.8–3.0)	0.16	1.5 (0.7–3.1)	0.32	1.2 (0.7–2.2)	0.54	0.7 (0.3–1.6)	0.47
T-Classification								
T1	Ref.				Ref.			
T2	0.8 (0.2–2.9)	0.74	0.6 (0.1–2.9)	0.55	1.3 (0.4–4.6)	0.66	1.3 (0.3–6.2)	0.71
T3	0.6 (0.2–1.7)	0.32	0.6 (0.2–2.0)	0.42	0.8 (0.3–2.5)	0.75	1.0 (0.3–3.6)	0.98
T4	0.9 (0.4–2.2)	0.78	0.6 (0.2–1.8)	0.39	1.7 (0.6–4.4)	0.31	1.6 (0.5–5.0)	0.43
N-Classification								
N0	Ref.				Ref.			
N1	0.5 (0.1–2.7)	0.45	1.1 (0.2–6.6)	0.92	0.6 (0.1–2.4)	0.45	0.6 (0.1–2.9)	0.56
N2	1.4 (0.5–3.8)	0.51	2.0 (0.7–6.2)	0.21	0.9 (0.4–2.4)	0.90	0.9 (0.3–2.4)	0.78
N3	1.4 (0.5–3.9)	0.51	2.8 (0.8–10.0)	0.11	1.5 (0.6–3.9)	0.41	1.8 (0.6–5.5)	0.30
Start during cycle								
Early start	Ref.				Ref.			
Late start	0.9 (0.5–1.7)	0.69	0.9 (0.4–2.0)	0.74	0.9 (0.5–1.6)	0.65	1.0 (0.5–2.1)	0.94
Very late start	0.3 (0.1–1.4)	0.12	0.2 (0.0–1.1)	0.06	0.5 (0.2–1.4)	0.19	0.3 (0.1–1.1)	0.06

**Table 8:** Analysis of prognostic factors influencing overall survival and progression-free survival since thoracic irradiation in ED-SCLC

Characteristics	OS since first irradiation				PFS since first irradiation			
	Univariate		Multivariate		Univariate		Multivariate	
	HR (95% CI)	*p*	HR (95% CI)	*p*	HR (95% CI)	*p*	HR (95% CI)	*p*
Sex								
Male	Ref.				Ref.			
Female	1.1 (0.6–1.8)	0.75	1.1 (0.6–2.0)	0.83	1.0 (0.6–1.7)	0.92	1.1 (0.6–2.0)	0.74
Age	1.0 (1.0–1.0)	0.10	1.0 (1.0–1.0)	0.58	1.0 (1.0–1.0)	0.61	1.0 (1.0–1.0)	0.70
Karnofsky								
80–100	Ref.				Ref.			
< 80	3.2 (1.8–5.6)	< 0.001	2.7 (1.4–5.5)	0.005	2.2 (1.3–3.7)	0.004	1.9 (1.0–3.7)	0.05
T-Classification								
T1	Ref.				Ref.			
T2	1.0 (0.3–3.6)	0.94	2.0 (0.4–9.4)	0.37	0.9 (0.3–3.2)	0.87	1.1 (0.3–4.3)	0.94
T3	1.5 (0.5–4.8)	0.49	2.0 (0.5–8.4)	0.32	1.3 (0.4–4.0)	0.64	1.3 (0.4–4.3)	0.72
T4	1.2 (0.4–3.4)	0.71	2.0 (0.5–7.2)	0.30	1.2 (0.4–3.5)	0.69	1.2 (0.4–3.6)	0.80
N-Classification								
N0	Ref.				Ref.			
N1	0.4 (0.0–3.7)	0.39	0.5 (0.0–8.2)	0.66	0.3 (0.0–2.8)	0.27	0.2 (0.0–2.2)	0.17
N2	2.5 (0.3–19.1)	0.37	2.4 (0.2–28.6)	0.48	2.2 (0.3–16.3)	0.46	1.2 (0.1–12.3)	0.90
N3	1.2 (0.2–8.6)	0.88	1.3 (0.1–17.0)	0.82	1.4 (0.2–10.0)	0.76	0.8 (0.1–8.6)	0.84
Start during cycle								
Early start	Ref.				Ref.			
Late start	0.2 (0.1–0.8)	0.02	0.2 (0.0–1.3)	0.09	0.5 (0.2–1.7)	0.29	1.1 (0.3–4.3)	0.88
Very late start	0.6 (0.2–1.4)	0.22	0.5 (0.2–1.8)	0.31	1.0 (0.4–2.1)	0.91	1.3 (0.5–3.5)	0.54

.
